# Recent Advances in Research of Plant Virus Movement Mediated by Triple Gene Block

**DOI:** 10.3389/fpls.2012.00276

**Published:** 2012-12-12

**Authors:** Andrey G. Solovyev, Natalia O. Kalinina, Sergey Y. Morozov

**Affiliations:** ^1^Belozersky Institute of Physico-Chemical Biology, Moscow State UniversityMoscow, Russia

**Keywords:** plant virus, virus movement, movement protein, triple gene block, TGB

## Abstract

The aim of this short review was to summarize recent advances in the field of viral cell-to-cell movement mediated by the triple gene block (TGB). The growing body of new research has uncovered links between virus cell-to-cell trafficking and replication, silencing suppression, virus spread over the plant, as well as suggested the roles of nucleus/nucleolus in plant virus transport and revealed protein-membrane associations occurring during subcellular targeting and cell-to-cell movement. In this context, our review briefly summarized current views on several potentially important functions of TGB proteins and on the development of new experimental systems that improved understanding of the molecular events during TGB-mediated virus movement.

## Introduction

**Figure 1:**
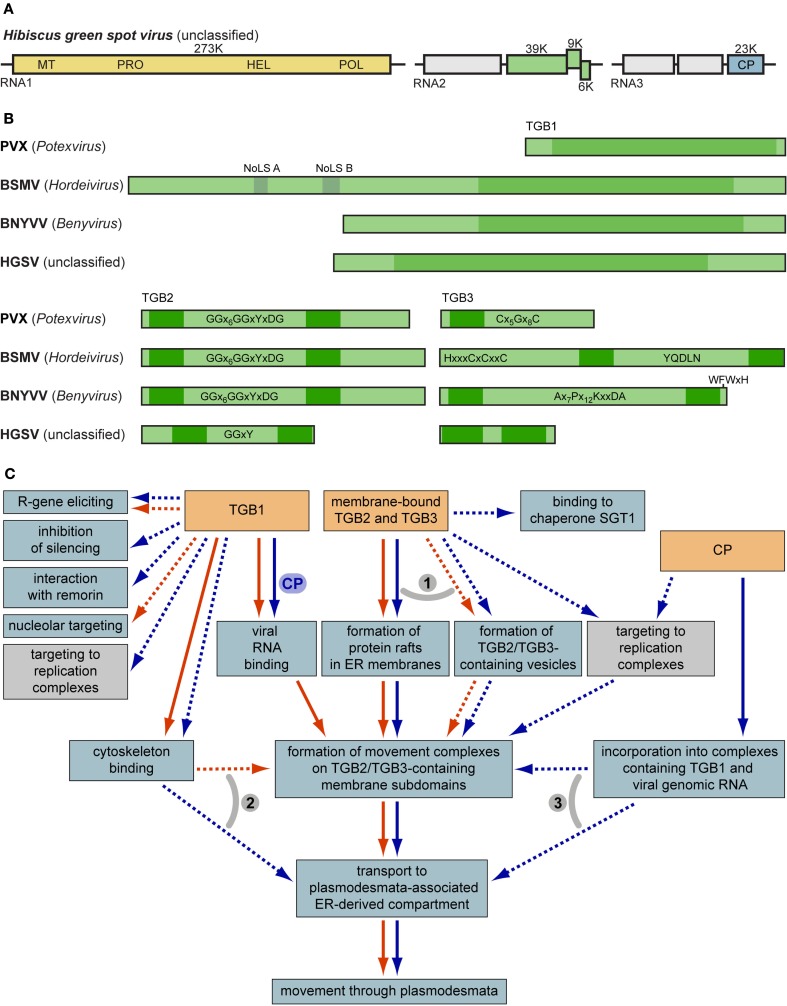
**(A)** Genome organization of the new TGB-containing virus HGSV. Boxes represent genome-encoded open reading frames. Replicase gene domains are shown in the yellow box: MT, methyltransferase; PRO, protease; HEL, RNA helicase; POL, RNA-dependent RNA polymerase. Green boxes represent the TGB. Blue box specifies the viral coat protein (CP). **(B)** Molecular organization of TGB1, TGB2, and TGB3 proteins. Nucleolar localization sequences and helicase domain regions of TGB1 are shown above the BSMV TGB1. Characteristic signature sequences in TGB2 and TGB3 are shown. Dark green boxes indicate hydrophobic transmembrane sequence segments. **(C)** General scheme of TGB-mediated intracellular movement and interactions of macromolecules. Processes specific for potex-like and hordei-like TGBs are shown by blue and red arrows, respectively. Note that the box ‘binding to chaperone SGT1’ means a functional interaction between TGB3 and SGT1 (Ye et al., 2012). Transport steps common for both potex- and hordei-like TGBs are shown by parallel arrows. Processes that are not proved to be involved directly in virus cell-to-cell movement are shown by dashed arrows. Numbered gray arcs indicate alternative pathways of intracellular trafficking. (1) TGB2 and TGB3 may travel to their destinations in specific membrane containers such as vesicles formed in a COPII-independent manner, or ER-specific membrane rafts (Verchot-Lubicz et al., 2010; this review). (2) Trafficking to the cell periphery of the TGB1 protein (and TGB1-containing RNPs) may exploit the cytoskeleton-based pathway with the immediate movement to PD-associated compartment, or via binding to TGB2/TGB3-containing membrane subdomains involved in cytoskeleton-dependent transport (Verchot-Lubicz et al., 2010; this review). (3) TGB2/3-specific membrane containers may bind movement-competent RNPs containing TGBp1. On the other hand, these complexes may be delivered directly to the neck region of PD through interactions with cytoskeleton (see above; Verchot-Lubicz et al., 2010; this review). For further details, see text.

In recent years, the molecular mechanism of triple gene block (TGB)-mediated cell-to-cell movement of plant viruses was extensively studied and reviewed (Morozov and Solovyev, 2003; Verchot-Lubicz et al., 2010; Hyun et al., 2011; Niehl and Heinlein, 2011; Schoelz et al., 2011; Torrance et al., 2011). Three overlapping TGB genes encode proteins designated TGB1, which contains the domain of RNA helicase of superfamily 1, TGB2 and TGB3, which are small membrane-associated proteins. Our previous reviews focused on common and distinct properties of two major classes of TGB modules, potex-like and hordei-like TGBs (Morozov and Solovyev, 2003; Verchot-Lubicz et al., 2010). The TGB2 protein is highly conserved in both TGB classes, whereas the structural properties of TGB1 and TGB3 proteins differ considerably between potex-like and hordei-like TGBs (Figure 1; Morozov and Solovyev, 2003).

The analysis of recently published sequences of new TGB-containing viruses allowed us to reveal two additional TGB classes, one included the TGB of *Beet necrotic yellow vein virus* (BNYVV) and several related viruses belonging to the unassigned genus *Benyvirus*, and the other was TGB of bacilliform *Hibiscus green spot virus* (HGSV; Figure 1A; Morozov and Solovyev, 2012). Similar to the hordei-like TGB3 proteins, the BNYVV TGB3 has two transmembrane domains. However, the BNYVV TGB3 protein differs from hordei-like proteins by the N-terminal transmembrane domain located close to the protein terminus and a conserved sequence signature found only in the genus *Benyvirus* (Morozov and Solovyev, 2003, 2012; Figure 1B). The HGSV TGB1 helicase is very distantly related to other TGB1 proteins and shows more similarity to the superfamily 1 replicative helicases of the genus *Benyvirus*; and HGSV TGB3 contains two long hydrophobic segments with extremely short central hydrophilic region and no similarity to any of three other groups of TGB3 proteins (Figure 1B; Melzer et al., 2012). Moreover, despite the highest conservation of TGB2 among hordei-, beny-, and potex-like TGB proteins (Morozov and Solovyev, 2003), the HGSV protein has the central hydrophilic segment only distantly related to other TGB2 proteins (Morozov and Solovyev, 2012; Figure 1B). The relation of the TGB1 protein to the replicative helicases of alpha-like positive-stranded RNA viruses (Koonin and Dolja, 1993), occurence of two helicase domains in the RNA replicase of an endornavirus (Koonin and Dolja, 2012) and the ability to suppress RNA silencing observed for helicase domains of viral replicases as well as TGB1 proteins (Bayne et al., 2005; Senshu et al., 2009, 2011) allowed us to put forward the hypothesis of a multi-step TGB evolution (Morozov and Solovyev, 2012).

In this review, only three directions of TGB research where considerable progress has been achieved in recent years were selected for detailed discussion. The advances in functional analysis of TGB-mediated virus movement are summarized in Table 1 and Figure 1C.

**Table 1:** **Overview of recent achievements in the cell biology studies of TGB**.

Research directions	Novel advances	Reference
TGB-mediated silencing suppression and virus movement	Five different potexviruses exhibit strong variations in the ability to suppress RNA silencing in *Nicotiana benthamiana*	Senshu et al. (2009)
	A specific mutation in potexvirus TGB1 significantly reduces the TGB1 silencing suppression ability but retains the protein movement functions unaffected	Lim et al. (2010a,b)
	Carlavirus TGB1 suppresses systemic RNA silencing in *Nicotiana benthamiana*	Senshu et al. (2011)
	PVX is able to infect triple dicer and AGO2 mutants of the non-host plant *Arabidopsis thaliana*	Jaubert et al. (2011)
	PVX TGB1 protein interacts with and destabilizes AGO1	Chiu et al. (2010)
Localization of the TGB1 protein in the nucleus and nucleolus	Potexvirus TGB1 proteins are shown to localize partly in the nucleus and nucleolus	Samuels et al. (2007), Lim et al. (2010b)
	The isolated N-terminal fragments of hordei- and pomovirus TGB1 can partly localize to the nucleolus	Wright et al. (2010), Semashko et al. (2012a)
	Hordeivirus TGB1 interacts with nucleolar proteins fibrillarin and coilin	Semashko et al. (2012a,b)
Protein-membrane association in the TGB-mediated intracellular movement	Potexvirus TGB3 exhibits the affinity to highly curved subdomains of cortical ER	Lee et al. (2010), Wu et al. (2011)
	Potexvirus TGB3 protein is targeted to membrane bodies at the cell periphery of yeast and plant cells and directs the TGB2 protein to these structures	Wu et al. (2011)
	TGB3 multimer formation is required for the transport to specific peripheral compartments	Lee et al. (2010)
	Protein regions necessary for the multimerization and subcellular targeting are mapped in both potex- and hordeivirus TGB3 proteins	Wu et al. (2011), Shemyakina et al. (2011), Sun and Zhang (2012)
Interaction of the TGB proteins with the cytoskeleton	Pomovirus TGB1 interacts with microtubules, and this interaction is not required for virus movement	Wright et al. (2010), Torrance et al. (2011)
	Hordeivirus TGB1 interacts with microtubules, and this interaction is involved in protein trafficking to plasmodesmata and aggresomes	Shemyakina et al. (2011)
	Assembly of potexvirus TGB1 rod-like inclusions depends on actin microfilaments but not on microtubules	Yan et al. (2012)
	The potexvirus TGB1 protein remodels host actin	Tilsner et al. (2012)
	Actin cytoskeleton is important for BSMV cell-to-cell movement and for localization of TGB3	Lim et al. (2012)
Association of the TGB proteins with the sites of virus replication	The potexvirus TGB3 protein is co-localized with the viral replicase in the ER	Bamunusinghe et al. (2009)
	Potexvirus TGB1 is responsible for virus genome compartmentalization in infected cells	Tilsner et al. (2012)
The role of the virus coat protein in potexvirus movement	The interaction between the potexvirus replicase and the coat protein is critical for virus movement in plant hosts	Lee et al. (2011)
	Potexvirus CP mutants deficient in the interaction with TGB1 can form virus particles but is unable to move in plant tissues	Tilsner et al. (2012)
	The N-terminal region of the PlAMV potexvirus coat protein is required for cell-to-cell movement but is dispensable for virion assembly	Ozeki et al. (2009)
Genomic cis-elements involved in TGB-mediated movement	The stem-loop structure in the 5′-terminal region of potexvirus RNA controls viral movement by interacting with the several host proteins and the virus coat protein	Cho et al. (2012a,b,c)
TGB-induced ER stress	PVX TGB3 induces unfolded protein response	Ye et al. (2011), Ye and Verchot (2011)
	Hordeivirus TGB3 overexpression induces severe changes of endomembrane system	Solovyev et al. (2012), Lim et al. (2012)
Eliciting of hypersensitive response	Hordeivirus TGB1 elicits hypersensitive response and binds host Bsr1 R-protein	Cui et al. (2012), Lee et al. (2012)
TGB3 upregulates host chaperones	PVX TGB3 upregulates ER resident and ubiquitin ligase chaperones	Ye et al. (2011, 2012), Ye and Verchot (2011)
TGB1 and remorin	PVX TGB1 protein binds to plant membrane raft protein remorin. This interaction impairs cell-to-cell movement of the virus	Raffaele et al. (2009), Perraki et al. (2012)

## TGB-Mediated Silencing Suppression and Virus Movement

The pioneering work of Bayne et al. (2005) proposed that the virus movement depends on multiple functions including silencing suppression. The idea that silencing suppression mediated by the *Potato virus X* TGB1 protein could be required for cell-to-cell PVX movement came from the finding that the movement function of some TGB1 mutants could be restored by the heterologous silencing suppressors P19 and HcPro provided *in trans*. However, at least one of the other functions of the potex-like TGB1 protein (the movement function *per se*) is essential for virus movement, since several TGB1 protein mutants are movement-defective but fully competent as silencing suppressors, and strong silencing suppressors could not support movement of such TGB1-deficient PVX (Bayne et al., 2005). In accordance with these data, a specific mutation in TGB1 of *Alternanthera mosaic virus*, another potexvirus, proved to significantly reduce the TGB1 silencing suppression ability but retained the protein movement functions unaffected (Lim et al., 2010a). A similar effect of this particular TGB1 point mutation on silencing suppression is observed in other potexviruses too (Lim et al., 2010b).

Five different potexviruses exhibit strong variations in the ability to suppress RNA silencing in *Nicotiana benthamiana*, and these variations result from the differences in the suppressor activities of their TGB1 proteins (Senshu et al., 2009). Moreover, recent data demonstrate that some of the potexvirus TGB1 proteins suppress both intracellular silencing and the silencing spread through the plant, while others such as TGB1 encoded by potexvirus PVX and *Potato virus M*, a carlavirus, mainly suppress the spread of the silencing signal (Voinnet et al., 2000; Senshu et al., 2011). The clue to understanding the drastic functional differences observed in *N. benthamiana* for TGB1 proteins encoded by viruses with different natural hosts was made in the study where PVX, which is not competent for movement in *Arabidopsis thaliana*, was able to infect the *A. thaliana* triple dicer mutant (*dcl2*, *dcl3*, and *dcl4*). Moreover, the restriction of PVX systemic movement on *A. thaliana* also depended on AGO2 (RNAse H-like Argonaute protein; Jaubert et al., 2011). Thus, the ability of PVX to infect the *Arabidopsis* triple dicer mutant and AGO2 mutant suggests that the PVX TGB1 protein does not function as an effective silencing suppressor in this host (Alvarado and Scholthof, 2009; Jaubert et al., 2011). The specificity of TGB1 interaction with host and non-host proteins is one of the explanations of this phenomenon. Indeed, previous reports have shown that several viral silencing suppressors directly target AGO proteins and either prevent siRNA loading or induce AGO degradation (Alvarado and Scholthof, 2009; Csorba et al., 2010; Burgyán and Havelda, 2011; Shimura and Pantaleo, 2011; Schott et al., 2012). Recently, the PVX TGB1 protein was also reported to interact with AGO1, AGO2, AGO3, and AGO4 and destabilize AGO1 (Chiu et al., 2010).

Recently, Duan et al. (2012) demonstrated that the interaction of silencing suppressor 2b encoded by cucumoviruses with AGO proteins *in vivo* was required, in addition to the suppression function itself, for the nucleolar targeting of 2b and contributed to the re-distribution of both the 2b and AGO proteins in the nucleus. Therefore, TGB1 can be expected to be capable of trafficking to the nucleus and nucleolus. Indeed, the TGB1 proteins encoded by two potexviruses, *Alternanthera mosaic virus* and *Narcissus mosaic virus*, were shown to localize partly in the nucleus and nucleolus, and their nucleolar localization was experimentally proved to be essential for the efficient suppression of RNA silencing, probably through TGB1 interaction with nucleolar components of the host RNA silencing machinery (Lim et al., 2010b). However, the PVX TGB1 protein was localized to the nucleus but not to the nucleolus (Samuels et al., 2007).

## Possible Link between the TGB1 Nucleolar Localization and Virus Long-Distance Movement

An increasing number of reports reveals that the proteins of many RNA viruses localize to the nucleus and its sub-compartments (mainly, to the nucleolus and the Cajal bodies), interact with nuclear/nucleolar proteins and divert host protein functions in order to exert novel role(s) during virus infection (Hiscox, 2007; Greco, 2009; Taliansky et al., 2010).

Analysis of TGB1 amino acid sequences employing the web service NoD, the nucleolar localization sequence detector (Scott et al., 2011), reveals nucleolar localization signals (NoLS) neither in HGSV, PVX, and other potex-like TGB1 proteins, nor in benyvirus-encoded proteins (our unpublished observations). On the other hand, all analyzed hordei-like TGB1 proteins are predicted to possess at least one NoLS: two NoLS sequences (NoLS A and B) were found in the proteins of all hordeiviruses, while a single NoLS was predicted in the pomovirus and pecluvirus proteins (Figure 1B). Therefore, we propose that the ability of the potexvirus TGB proteins to localize to the nucleolus can be due to their interactions with cell proteins (see above), whereas the transport of hordeivirus and pomovirus TGB1 proteins to the nucleolus can be directed by their own targeting signals.

In agreement with the NoLS predictions in hordei-like TGB1 proteins, several reports demonstrate the nuclear/nucleolar targeting of pomovirus and hordeivirus GFP-tagged TGB1 proteins observed along with their cytoplasmic localization (Wright et al., 2010; Semashko et al., 2012a). The NoLSs in TGB1 proteins encoded by viruses of both genera were predicted in the unstructured N-terminal domain (NTD; Figure 1B), which is present in all hordei-like TGB1 proteins (Makarov et al., 2009). These predictions are validated by the observation that the isolated N-terminal fragments of TGB1 can partly localize to the nucleolus (Wright et al., 2010; Semashko et al., 2012a). Moreover, mutations of basic residues in this region of the hordei-like pomovirus TGB1 protein abolish its nucleolar accumulation (Torrance et al., 2011). Similarly, mutagenesis of the basic amino acid residues in predicted hordeivirus NoLS A (aa107-136) and B (aa171-194) reveal that these protein regions are indeed involved in the protein localization to the nucleolus (Semashko et al., 2012a).

The hordeivirus TGB1 protein is able to bind fibrillarin, the major nucleolar protein, *in vitro*. The interaction of the two proteins, which involves the glycine-arginine-rich domain of fibrillarin and the 82 N-terminal amino acid residues of TGB1 protein, can also be detected by bimolecular fluorescence complementation upon transient coexpression in *N. benthamiana* plants (Semashko et al., 2012a). Additionally, the TGB1 NTD of two hordeiviruses is able to interact *in vitro* and *in vivo* with coilin, the major structural component of Cajal bodies, the subnuclear structures revealed in nuclei of many eukaryotes, including plants; and substitutions in the NoLS A resulted in an almost complete loss of the NTD ability to bind coilin (Semashko et al., 2012b; Kalinina and Guseinov, unpublished results). Fibrillarin is known to interact with the umbravirus ORF3 protein in the nucleolus, and this complex re-locates from the nuclei to the cytoplasm and takes part in the formation of viral cytoplasmic ribonucleoproteins (RNPs), which are capable of long-distance movement (Taliansky et al., 2010). In the hordeivirus and pomovirus TGB1 proteins, positively charged motifs corresponding to NoLS proved to be dispensable for the virus transport from cell-to-cell but necessary for the long-distance virus movement (Kalinina et al., 2001; Wright et al., 2010; Torrance et al., 2011). Collectively, these data suggest that, unlike viruses with the potex-like TGB where the nuclear localization of the TGB1 protein is due to its functions in suppression of RNA silencing, the localization of hordei-like TGB1 to the nucleus/nucleolus may result from its functions in virus long-distance movement. We hypothesize that this difference can be explained by different structure of the transport form of the viral genome in viruses with potex-like and hordei-like TGB, namely, the TGB1-modified virions in the former group and TGB1-formed non-virion RNPs in the latter one (Verchot-Lubicz et al., 2010). Presumably, the formation of transport-competent TGB1-containing virions does not require functions of cell nucleolar protein(s).

## Protein-Membrane Association in the TGB-Mediated Intracellular Movement

Transport of the TGB1 protein to plasmodesmata is generally accepted to require the functions of the TGB2 and TGB3 proteins (Verchot-Lubicz et al., 2010). Previous data clearly demonstrated that the TGB3 protein contains signals of intracellular transport at least in viruses with the hordei-like TGB (Tilsner et al., 2010; Shemyakina et al., 2011; Sun and Zhang, 2012). Being capable of interaction with other TGB proteins, the TGB3 protein serves as a “driving force” of their intracellular transport to plasmodesmata-associated sites (Zamyatnin et al., 2004; Lim et al., 2008, 2009). Therefore, understanding the mechanism of TGB3 protein translocation from sites of its synthesis to plasmodesmata is of key importance for unraveling the details of the intracellular phase of TGB-mediated transport (Figure 1C).

In yeast cells, the behavior of the TGB3 protein encoded by *Bamboo mosaic virus* (BaMV), a potexvirus, is similar to that in plant cells: the protein is able to be targeted to the membrane bodies at the cell periphery and to direct the TGB2 protein to these structures (Lee et al., 2010). As in plant cells, the peripheral membrane TGB3-containing structures in yeast cells represent a subdomain of the cortical ER (Lee et al., 2010; Wu et al., 2011). Moreover, the TGB3-containing structures in yeast cells reside within discrete cortical ER regions enriched in cell reticulons Rtn1 and Yop1. These proteins belong to two families of integral ER membrane proteins necessary for the formation of highly curved membrane tubules of cortical ER in eukaryotic cells (Lee et al., 2010). The potexvirus TGB3 protein co-localized with a plant-encoded Rtn1-related protein in tobacco leaf cells as well, thus, validating the data obtained in yeast cells (Lee et al., 2010). Importantly, the desmotubule, an ER tubule, which locates in plasmodesmata and interconnects ER networks in neighboring cells, is extremely narrow and therefore has a high membrane curvature. Tilsner et al. (2011) recently suggested that the Rtn1- and Yop1-related proteins are required for the formation and stabilization of desmotubule, while the TGB3 protein can exhibit a high affinity to this specific plasmodesmal sub-structure. Indeed, the hordei-like TGB3 proteins of BSMV and *Potato mop-top virus* (PMTV) were shown to be retained within cell wall-embedded structures upon plasmolysis (Lim et al., 2009; Tilsner et al., 2010), which supports the hypothesis of their localization to the desmotubule.

As demonstrated for both potex-like and hordei-like TGBs, the TGB3 protein trafficking to plasmodesmata-associated membrane structures is COPII-independent and, thus, employs an unconventional mechanism, which does not involve the exit from ER in COPII-transport vesicles (Figure 1C; Schepetilnikov et al., 2005, 2008; Lee et al., 2010). The COPII-independent TGB3-specific trafficking to plasmodesmata-associated peripheral ER compartments requires specific signals in the TGB3 sequence. It was demonstrated that the targeting of hordei-like TGB3 protein was determined by a composite signal comprising the highly conserved sequence motif YQDLN located in the central hydrophilic protein region and the C-terminal transmembrane domain (Schepetilnikov et al., 2008; Tilsner et al., 2010; Lim et al., 2012). Recent studies show that these TGB3 regions play distinct roles. Analyses of the hordeivirus TGB3 protein demonstrate that the true signal of its intracellular transport resides in the protein C-terminal transmembrane segment, while the YQDLN motif is involved in protein oligomerization, which is essential for the functioning of targeting signal (Shemyakina et al., 2011; Sun and Zhang, 2012). Therefore, the hordeivirus TGB3 protein with the functional C-terminal targeting signal is able to enter its specific translocation pathway only in the form of multimeric complexes. Such TGB3-containing complexes represent the natural form of this protein found in hordeivirus-infected tissue (Shemyakina et al., 2011).

The residues responsible for specific targeting and self-interaction have been recently mapped in the BaMV TGB3 protein. The targeting to Rtn1/Yop-enriched cortical ER subdomains requires the C-terminal hydrophilic protein region, specifically, several critical residues conserved in the TGB3 proteins encoded by different potexviruses (Wu et al., 2011). Therefore, the functionally equivalent transport signals identified in the hordeivirus TGB3 protein (the transmembrane sequence domain) and the potexvirus TGB3 protein (the hydrophilic sequence region) are strikingly different in their properties. The potexvirus TGB3 is capable of multimer formation, and the residues involved in protein self-interaction were mapped to the TGB3 region containing the protein sorting signal (Wu et al., 2011). It should be emphasized that, similarly to the hordeivirus TGB3 protein, the potexvirus TGB3 protein self-interaction is a pre-requisite for its correct subcellular targeting (Shemyakina et al., 2011; Wu et al., 2011).

The mechanism of TGB3 intracellular transport was hypothesized to involve either lateral translocation of TGB3-formed rafts, which also incorporate the TGB2 protein, in the plane of the ER membranes (Morozov and Solovyev, 2003; Wu et al., 2011) as postulated for the *Tobacco mosaic virus* MP (Epel, 2009), or vesicles of unknown nature tightly associated with the cortical tubular ER as observed for the PVX and PMTV TGB3 proteins (Samuels et al., 2007; Verchot-Lubicz et al., 2010).

Thus, the new data clearly show that the potex-like and hordei-like TGB3 proteins, which have markedly different structure (Morozov and Solovyev, 2003), nevertheless exhibit similar functional properties (Figure 1C) including the abilities for multimerization and multimerization-dependent subcellular targeting. Similar to hordeivirus TGB3 proteins, the BaMV TGB3 protein is found in discrete membrane bodies located at the cell periphery corresponding to highly curved subdomains of cortical ER (Wu et al., 2011) and is able to interact with the TGB2 protein, therefore ensuring the TGB2 co-targeting to TGB3-containing structures (Lee et al., 2010; Wu et al., 2011). Another parallel between the potex-like and hordei-like TGBs is provided by the recently shown interaction between the BaMV TGB2 and TGB1 proteins (Wu et al., 2011). This finding suggests that the complex containing the two membrane proteins encoded by potex-like TGB may direct the TGB1 protein to plasmodesmata-associated sites (Figure 1C). This hypothesis agrees with the reported ability of the hordei-like PMTV TGB2/TGB3 proteins to target the respective TGB1 protein to peripheral membrane compartments and to the plasmodesmata interior (Zamyatnin et al., 2004) as well as with the observed interactions of the BSMV TGB3 protein with both TGB2 and TGB1 proteins (Lim et al., 2008). The new findings make it possible to propose a general model of intracellular transport for hodeivirus and potexvirus TGB proteins (Figure 1C). This model includes common and specific events for both types of proteins as well as possible alternative pathways of trafficking process.

## Conclusion

Studies carried out in the recent years reveal new aspects of the TGB-mediated virus movement, such as the accumulation of TGB3 protein in the cortical highly curved ER regions enriched in cell reticulons and involvement of the TGB1 protein in the interactions with the cellular RNA silencing machinery. The current research uncovers tight links between virus replication and cell-to-cell movement, the role of cytoskeleton, and the requirements for specific genomic RNA regions for TGB-mediated transport. In this short review we focused the reader’s attention on the three trends in TGB studies to inspire further progress in the field.

## Conflict of Interest Statement

The authors declare that the research was conducted in the absence of any commercial or financial relationships that could be construed as a potential conflict of interest.
